# Supplementary material for: Engineering potent live attenuated coronavirus vaccines by targeted inactivation of the immune evasive viral deubiquitinase
Source: Nat Commun. 2023 Feb 28;14:1141. doi: 10.1038/s41467-023-36754-z (PMC9973250; doi:10.1038/s41467-023-36754-z)
Supplement: Supplementary file 1 — Supplementary Information [file 41467_2023_36754_MOESM1_ESM.pdf]

Engineering potent live attenuated coronavirus vaccines by targeted inactivation of the immune evasive viral deubiquitinase

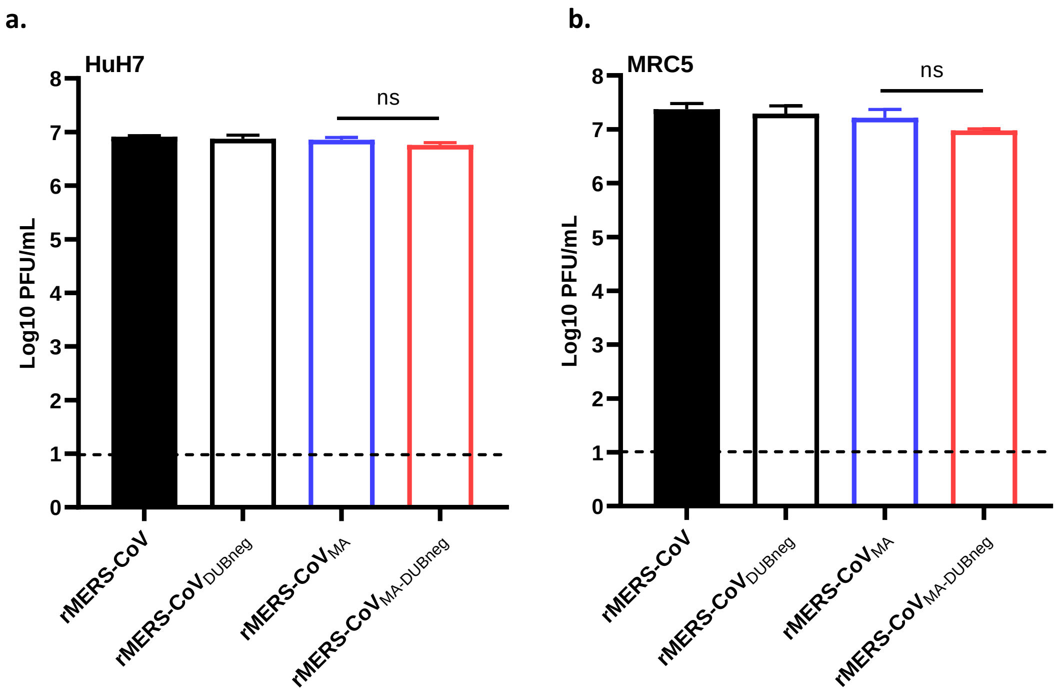

Supplementary Figure 1. Replication of DUB negative rMERS-CoV. (a) Huh7 or (b) MRC5 were infected with indicated MERS-CoV at MOI 1 and virus titers at 24 h.p.i were determined by plaque assay on Huh7 cells. Infections were performed in triplicate and error bars represent standard error of mean. Statistical significance between means (n = 3, independent replicates) was assessed by an unpaired two-tailed Student's t test. (NS: not significant). The limit of detection for infectious titer is 10 PFU/mL and is indicated with a dashed line. Source data are provided as a Source Data file.

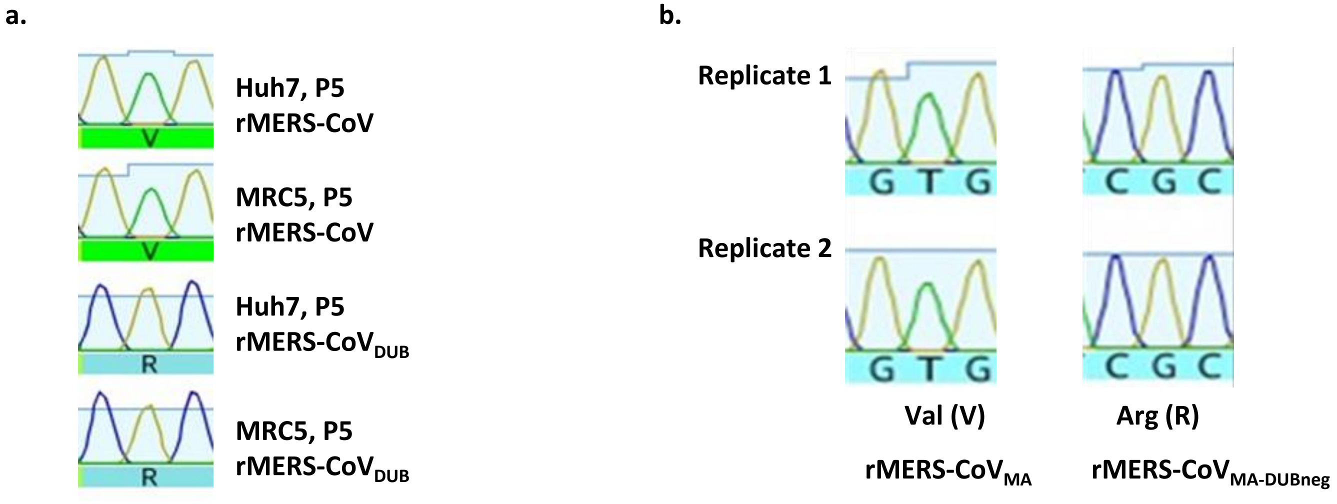

Supplementary Figure 2. Genetic stability of the DUB-negative rMERS-CoV *in vitro* and *in vivo*. In order to evaluate the genetic stability of the V1691R substitution in the rMERS-CoV PLpro coding region. rMERS-CoV was passaged 5 times in Huh7 and MRC5 cells and the PLpro coding region of the genome was sequenced by Sanger sequencing (a). After five passages, the presence of all engineered substitutions was confirmed, in the absence of any additional (unintended) substitutions (a). To confirm the genetic stability of the V1691R substitution *in vivo*, RNA was isolated from homogenized lung tissue collected 3 days p.i, and the PLpro coding was amplified by RT-PCR and sequenced (b). In the wt (rMERS-CoV$_{MA}$) virus-infected mice, the consensus sequence of the PCR product was found to be identical to the sequence of the BAC-based cDNA clone from which the rMERS-CoV$_{MA}$ had been launched (b). RNA isolated from mice infected with the DUB-negative rMERS-CoV$_{MA}$ and sequenced also revealed the presence of the V1691R substitution in PLpro without additional substitution in Plpro (b).

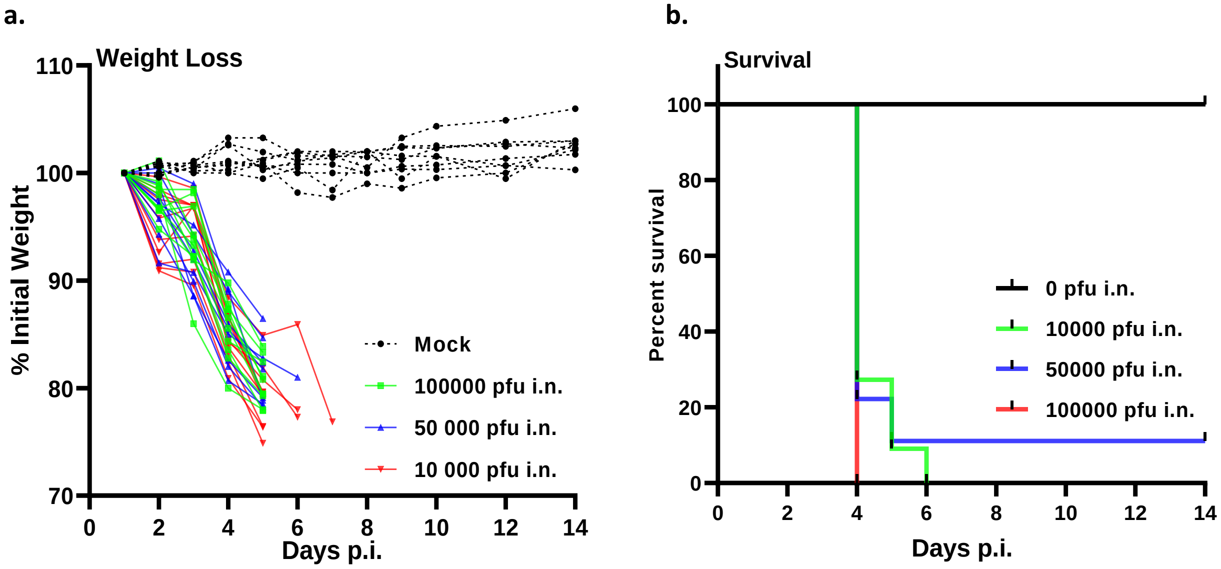

Supplementary Figure 3. rMERS-CoV$_{MA}$ caused dose-dependent lethal lung disease in hDPP4 KI mice. Groups of 9 or 10 (Mock/0 pfu: *n* = 9; all groups: *n* =10) mice were infected intranasally with rMERS-CoV$_{MA}$ with doses ranging from 10^4 to 10^5 pfu per animal. (a) Weight loss kinetics and (b) survival percentages over time were monitored for 14 days. A Log-Rank test was used for survival (****p<0.0001). Source data are provided as a Source Data file.

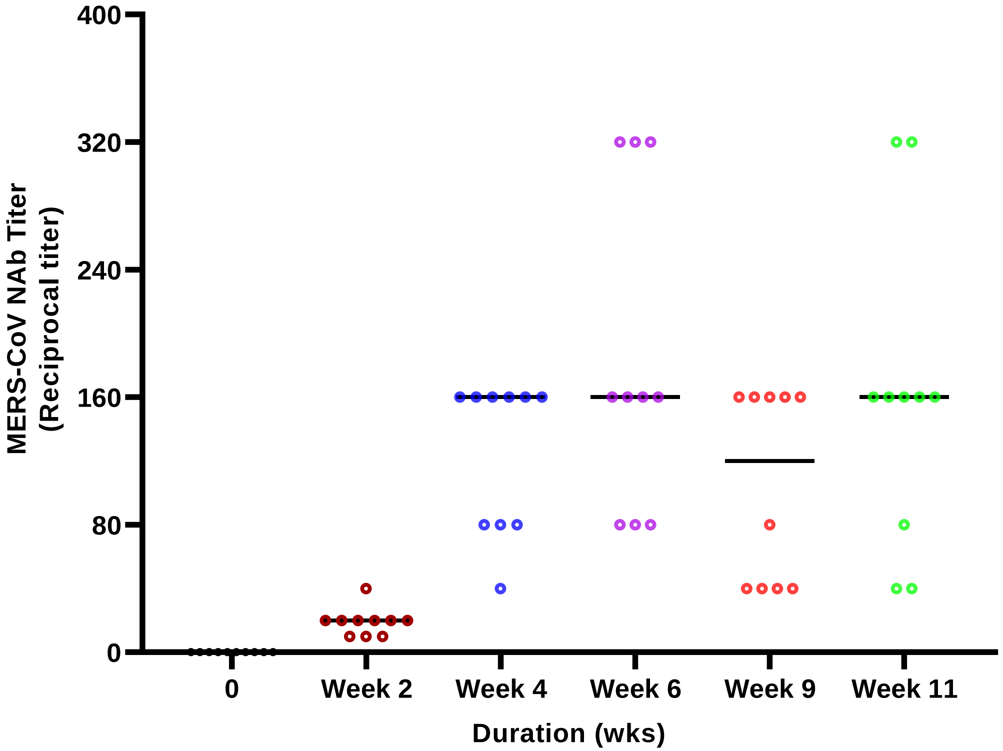

Supplementary Figure 4. DUB-negative MERS-CoV elicits potent and sustained neutralizing antibodies. Groups of 10 mice (*n* = 10) were immunized intranasally with either 10^4 pfu of the rMERS-$CoV_{MA\text{-}DUBneg}$ or mock immunized. At week 0, 2, 4, 6, 9 and 11, blood was collected from mice and twofold serially diluted serum samples were tested for neutralization activity against the rMERS-CoV_{MA} in Huh7 cells. Black horizontal lines indicate mean reciprocal titers and colored circles indicate individual values per group. Source data are provided as a Source Data file.

a.

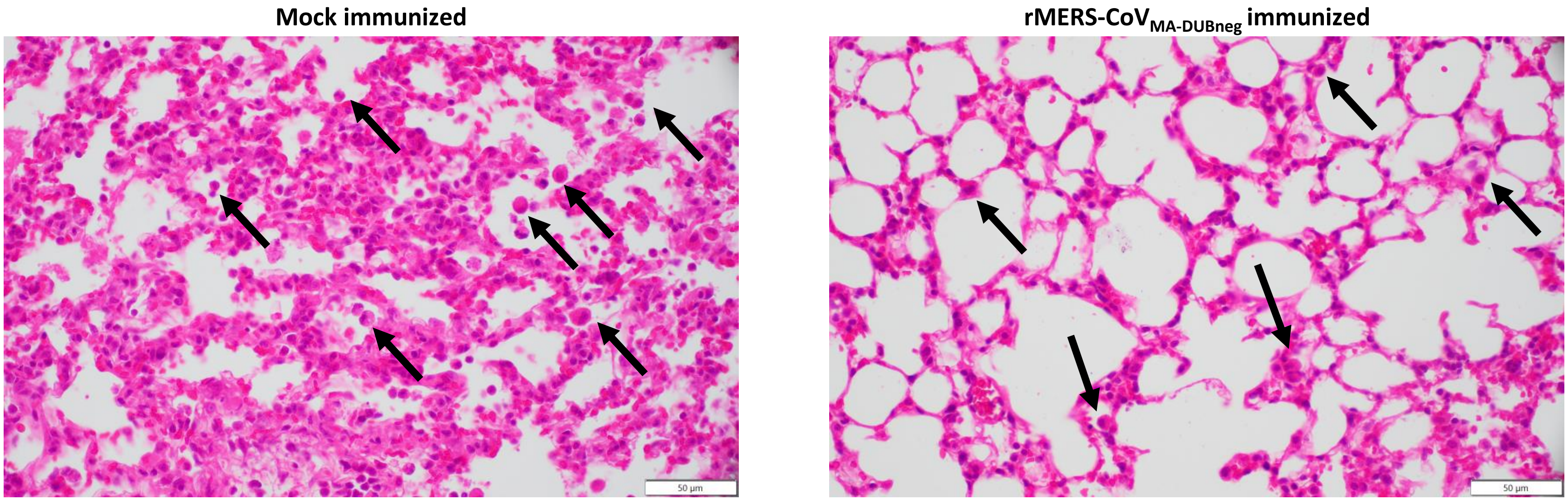

b.

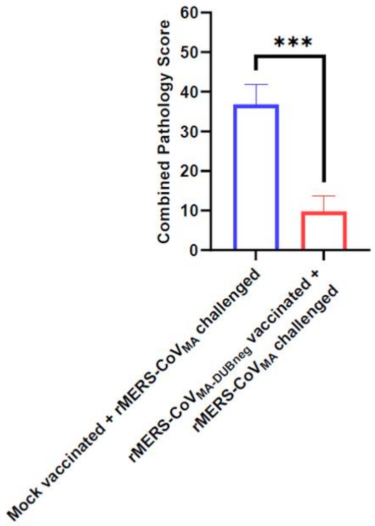

Supplementary Figure 5. Lung pathology of mock or DUB-negative rMERS-CoV$_{MA}$ immunized hDPP4 KI mice challenged with wildtype rMERS-CoV$_{MA}$ on day 4 post challenge.

a. Photomicrographs of representative lung lesions are shown from a mock (left panel) or a DUB-negative rMERS-CoV$_{MA}$ (right panel) immunized animal (H&E stain, 400x magnification). Moderate to high numbers of predominantly macrophages and fewer viable neutrophils are present in the expanded alveolar septa and alveolar spaces (arrows) in the lung of the mock immunized animal. In the lung of the rMERS-CoV$_{MA-DUBneg}$ immunized animal, however, these same inflammatory cells are less abundant and mostly limited to the alveolar septa (arrows).

a. Bar chart with semi-quantitative combined lung pathology scores. Bar heights indicate group means (n=4) and error bars the standard error of the mean. The difference between the mock and rMERS-CoV$_{MA-DUBneg}$ vaccinated groups was highly significant (***$P<0.001$, unpaired, two-tailed Student's t-test). Source data are provided as a Source Data file.

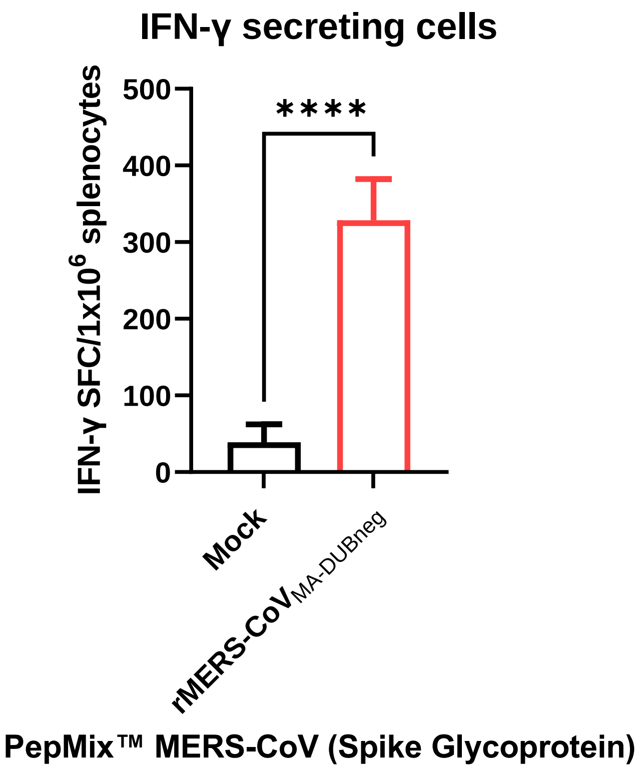

Supplementary Figure 6. Cellular immunogenicity of rMERS-CoV$_{\text{MA-DUBneg}}$ in hDPP4 KI mice. Human DPP4 KI mice were intranasally immunized with either 10^4PFU rMERS-CoV$_{\text{MA-DUBneg}}$ or mock immunized with DMEM (*n* = 6). At 4 weeks after immunization, IFN-γ ex vivo ELISPOT was performed on splenocytes. Bar heights indicate group means (*n* = 6) and error bars the standard error of the mean. The difference between the mock and rMERS-CoV$_{\text{MA-DUBneg}}$ vaccinated groups was significant (****P<0.0001, unpaired, two-tailed Student's t-test). Source data are provided as a Source Data file.

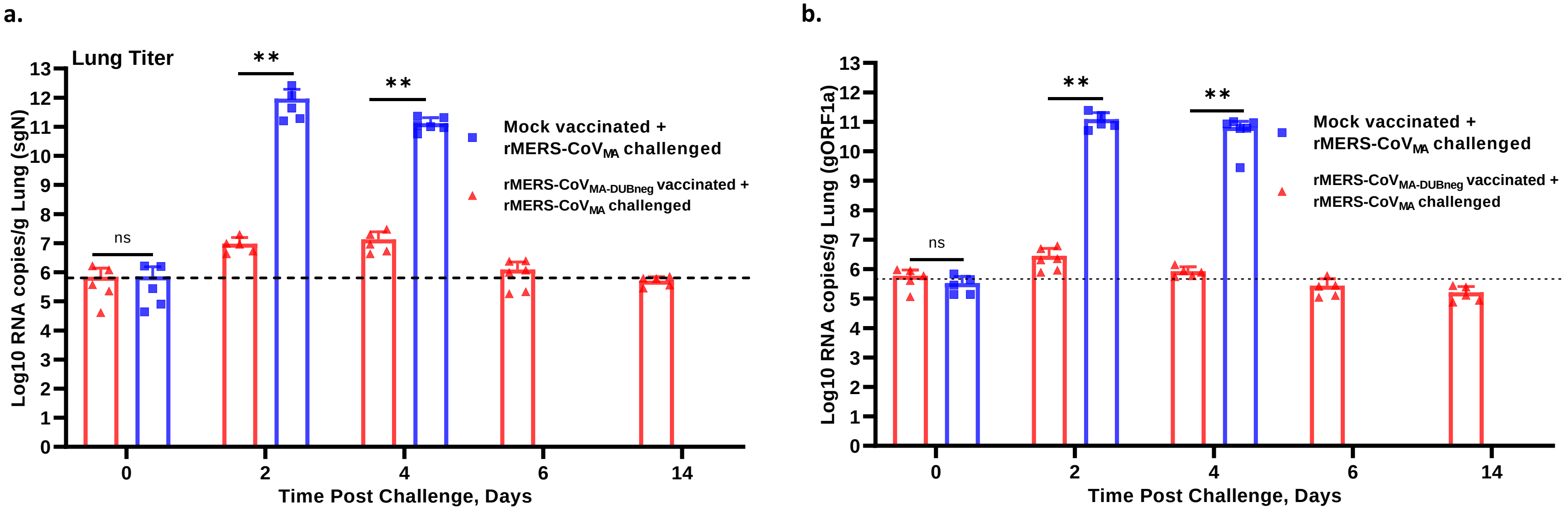

Supplementary Figure 7. Viral loads in the lung after challenge. Viral sub-genomic RNA (a) and genomic RNA (b) in the lungs of rMERS-CoV$_{MA}$ challenged mock vaccinated or DUB-negative rMERS-CoV$_{MA}$ hDPP4 KI mice on days 0, 2, 4, 6 and 14 post-challenge. PGK1 and lung weights were used to normalize the RNA copy levels. The mean per group (n = 5) and the virus RNA copies per gram of lung tissue are presented with symbols for each mouse and the error bars represent standard error of mean. Statistical significance between means at day 2 and 4 was assessed by an unpaired two-tailed Student's *t* test. a. day 0 (ns>0.999) , day 2 ($^{**}P < 0.0079$), day 4 ($^{**}P = 0.0025$) and b. day 0 (ns>0.2076) , day 2 ($^{**}P < 0.0089$), day 4 ($^{**}P = 0.0062$) . The limit of detection for RNA copies/g Lung is indicated with a dashed line. Source data are provided as a Source Data file.

Virus	NT mutation	AA mutation	Genetic region	Frequency, %
rMERS-CoV V1691R, Huh7, p10	T2514A	Y553N	nsp2	20.3
	C2544A	Q563K	nsp2	17.4
	A2646G	S597G	nsp2	11.7
	C5348T	Y837Y	nsp3	23.0
	G5351C	V838C	nsp3	12.1
	GTG5349-1AGC	V838S	nsp3	12.2
	GTG5349-1CAC	V838H	nsp3	8.2
	GTG5349-1CGC	**V838R**	nsp3	62.0
	C9631A	P378H	nsp4	12.3
	C10217T	F66F	nsp6	11.8
	A11503T	K189N	nsp6	10.2
	T20761C	**Y53Y**	2'-O-methyltransferase	100
	T24091C	I879T	Spike protein	13.7
	A26762C	I224L	NS4B protein	16.0
	G27162A	**W108***	NS5 protein	100
	G27634A	N15K	Envelope protein	11.3
rMERS-CoV WT, Huh7, p10	T20761C	**Y53Y**	2'-O-methyltransferase	99.8
	A24514G	Q1020R	Spike protein	69.5
	C24556T	S1034F	Spike protein	67.2
	G27162A	**W108***	NS5 protein	99.8

Supplementary Table 1. Genetic stability analysis of the DUB-negative rMERS-CoV (rMERS-CoV V1691R/V838R). Table summarizing the NGS data genetic stability analysis of the DUB-negative rMERS-CoV after 10 passages in Huh7 cells. Notations: introduced amino acid (AA) mutations shown with bold font; silent AA mutations are underlined; W108*, intended introduced stop codon at ORF5/NS5. Frequency of mutations was calculated after mapping of NGS reads to the reference sequence of MERS (RefSeq access number: NC_019843.3); mutations frequency for the region (5349-5351 nt) was obtained using NGS reads that covers completely the region (5301-5390 nt). NT position of mutation are shown according to the reference sequence, amino acid position of mutations are shown according to the position in translation of the corresponding CDS. Source data are provided as a Source Data file.

**Vaccination type**	**Extent**	**Alveolar interstitial inflammation**			**Perivascular mixed inflammatory cell infiltrate and edema**	**Necrosis**	**Intra-alveolar neutrophils**	**Intra-alveolar macrophages**	**Hyaline membranes per alveolus**	**Intra-alveolar fluid**	**Intra-alveolar hemorrhage**	**Alveolar septal thickening**	**Combined path score**
		**Neuts**	**Macs**	**Lymphocytes**									
**Mock**	**1,00**	**0,00**	**1,00**	**0,00**	**0,00**	**0,00**	**0,00**	**1,00**	**0,00**	**0,33**	**0,00**	**0,00**	**2,33±0,47**
Animal 1	1	0	1	0	0	0	0	1	0	0	0	0	**2**
Animal 2	1	0	1	0	0	0	0	1	0	0	0	0	**2**
Animal 3	1	0	1	0	0	0	0	1	0	1	0	0	**3**
**rMERS-CoV$_{MA}$**	**2,25**	**2,00**	**2,00**	**1,25**	**1,75**	**0,75**	**1,75**	**2,00**	**0,50**	**1,25**	**1,75**	**0,75**	**38,00±18,60**
Animal 4	1	2	2	1	1	0	1	1	0	0	2	0	**10**
Animal 5	2	2	2	2	2	1	2	2	0	1	2	1	**34**
Animal 6	3	2	2	1	2	1	2	3	1	3	2	1	**60**
Animal 7	3	2	2	1	2	1	2	2	1	1	1	1	**48**
**rMERS-CoV$_{MA\text{-}DUBneg}$**	**1,25**	**1,00**	**1,75**	**1,00**	**1,00**	**0,00**	**0,25**	**0,25**	**0,00**	**0,00**	**0,75**	**0,75**	**8,25±2,59**
Animal 8	1	1	2	1	1	0	1	1	0	0	1	1	**9**
Animal 9	1	1	2	1	1	0	0	0	0	0	1	1	**7**
Animal 10	1	1	1	1	1	0	0	0	0	0	1	0	**5**
Animal 11	2	1	2	1	1	0	0	0	0	0	0	1	**12**

Notes: Extent: 0=none/minimal (<5%), 1=focal (5-33%), 2=multifocal (33-66%), 3=diffuse (66-100%); Alveolar interstitial inflammation, perivascular mixed inflammatory cell infiltrates and edema, necrosis, intra-alveolar neutrophils, macrophages, and hemorrhage: 0=none, 1=mild, 2=moderate, 3=severe; Alveolar septal thickening: 0=none, 1=2-fold increased, 2=2-4-fold increased, 3=more than 4-fold increased compared with unaffected septa; Hyaline membranes, intra-alveolar proteinaceous fluid: 0=none, 1=1, 2=more than 1 per alveolus. Abbreviations: Neuts=neutrophils; Macs=macrophages; path=pathology; p.i.=post infection.

Supplementary Table 2. Lung pathology scores of mock, rMERS-CoV$_{MA,}$ and rMERS-CoV$_{MA\text{-}DUBneg}$ infected hDPP4 KI mice on day 4 post infection. Table listing all semi-quantitatively scored lung lesions per animal and the calculated combined lung pathology score. Group means (all scores) and standard error of the mean (combined score only) are indicated in bold. Source data are provided as a Source Data file.

Vaccination type	Extent	Alveolar interstitial inflammation			Perivascular mixed inflammatory cell infiltrate and edema	Necrosis	Intra-alveolar neutrophils	Intra-alveolar macrophages	Hyaline membranes per alveolus	Intra-alveolar fluid	Intra-alveolar hemorrhage	Alveolar septal thickening	Combined path score
		Neuts	Macs	Lymphocytes									
**Mock**	**3,00**	**1,25**	**3,00**	**1,00**	**1,75**	**0,50**	**0,50**	**1,00**	**1,00**	**0,50**	**0,00**	**1,50**	**36,75 ± 4,44**
Animal 12	3	1	3	1	2	1	1	1	1	1	0	1	**39**
Animal 13	3	1	3	1	2	0	1	1	1	1	0	1	**36**
Animal 14	3	2	3	1	2	1	0	1	1	1	0	2	**42**
Animal 15	3	1	3	1	1	0	0	1	1	0	0	2	**30**
**rMERS-CoV$_{MA\text{-}DUBneg}$**	**1,50**	**0,00**	**2,00**	**1,75**	**1,00**	**0,00**	**0,00**	**0,00**	**0,25**	**0,25**	**0,00**	**1,25**	**9,75 ± 3,34**
Animal 16	2	0	2	1	1	0	0	0	1	1	0	1	**14**
Animal 17	2	0	2	2	1	0	0	0	0	0	0	1	**12**
Animal 18	1	0	2	2	1	0	0	0	0	0	0	1	**6**
Animal 19	1	0	2	2	1	0	0	0	0	0	0	2	**7**

Notes: Extent: 0=none/minimal (<5%), 1=focal (5-33%), 2=multifocal (33-66%), 3=diffuse (66-100%); Alveolar interstitial inflammation, perivascular mixed inflammatory cell infiltrates and edema, necrosis, intra-alveolar neutrophils, macrophages, and hemorrhage: 0=none, 1=mild, 2=moderate, 3=severe; Alveolar septal thickening: 0=none, 1=2-fold increased, 2=2-4-fold increased, 3=more than 4-fold increased compared with unaffected septa; Hyaline membranes, intra-alveolar proteinaceous fluid: 0=none, 1=1, 2=more than 1 per alveolus. Abbreviations: Neuts=neutrophils; Macs=macrophages; path=pathology.

Supplementary Table 3. Lung pathology scores of DUB-negative rMERS-CoV$_{MA}$ or mock immunized hDPP4 mice challenged with wildtype rMERS-CoV$_{MA}$ on day 4 post challenge. Table listing all semi-quantitatively scored lung lesions per animal and the calculated combined lung pathology score. Group means (all scores) and standard error of the mean (combined score only) are indicated in bold. Source data are provided as a Source Data file.
