# Supplementary material for: Engineering potent live attenuated coronavirus vaccines by targeted inactivation of the immune evasive viral deubiquitinase
Source: Nat Commun. 2023 Feb 28;14:1141. doi: 10.1038/s41467-023-36754-z (PMC9973250; doi:10.1038/s41467-023-36754-z)
Supplement: Supplementary file 3 — Reporting summary [file 41467_2023_36754_MOESM3_ESM.pdf]

nature portfolio

Corresponding author(s): Sebenzile Myeni and Marjolein Kikkert
Last updated by author(s): Feb 12, 2023

Reporting Summary

Nature Portfolio wishes to improve the reproducibility of the work that we publish. This form provides structure for consistency and transparency in reporting. For further information on Nature Portfolio policies, see our Editorial Policies and the Editorial Policy Checklist.

Statistics

For all statistical analyses, confirm that the following items are present in the figure legend, table legend, main text, or Methods section.

n/a	Confirmed	
☐	☒	The exact sample size (*n*) for each experimental group/condition, given as a discrete number and unit of measurement
☐	☒	A statement on whether measurements were taken from distinct samples or whether the same sample was measured repeatedly
☐	☒	The statistical test(s) used AND whether they are one- or two-sided<br>*Only common tests should be described solely by name; describe more complex techniques in the Methods section.*
☒	☐	A description of all covariates tested
☒	☐	A description of any assumptions or corrections, such as tests of normality and adjustment for multiple comparisons
☐	☒	A full description of the statistical parameters including central tendency (e.g. means) or other basic estimates (e.g. regression coefficient) AND variation (e.g. standard deviation) or associated estimates of uncertainty (e.g. confidence intervals)
☐	☒	For null hypothesis testing, the test statistic (e.g. *F*, *t*, *r*) with confidence intervals, effect sizes, degrees of freedom and *P* value noted<br>*Give P values as exact values whenever suitable.*
☒	☐	For Bayesian analysis, information on the choice of priors and Markov chain Monte Carlo settings
☒	☐	For hierarchical and complex designs, identification of the appropriate level for tests and full reporting of outcomes
☒	☐	Estimates of effect sizes (e.g. Cohen's *d*, Pearson's *r*), indicating how they were calculated

Our web collection on statistics for biologists contains articles on many of the points above.

Software and code

Policy information about availability of computer code

Data collection	Automated ELISPOT reader (Bio-Sys GmbH, Germany)
Data analysis	NGS data analysis: quality control and trimmings of reads, Trimmomatic v0.36; mapping reads to the reference sequence (GenBank NC_019843.3), Samtools v1.7; variant calling: Bcftools v.1.7. Statistical analysis: GraphPad Prism 9.3.1. Raw NGS data were deposited in SRA (BioProject: PRJNA933107, accession numbers: SRR23379932, rMERS-CoV v1691R Huh7 p10; SRR23379933, rMERS-CoV WT Huh7 p10) and can be accessed at this link: https://eur03.safelinks.protection.outlook.com/?url=https%3A%2F%2Fdataview.ncbi.nlm.nih.gov%2Fobject%2FPRJNA933107%3Freviewer%3Dfpbmt3cnd6620pg1pjgjgtn1nr&data=05%7C01%7CI.Sidorov%40lumc.nl%7Cf297df6d0c34449bf9b308db0b545aa4%7Cc4048c4fdd544cbd80495457aacd2fb8%7C0%7C0%7C638116229519224723%7CUnknown%7CTWFpbGZsb3d8eyJWIjoiMC4wLjAwMDAiLCJQIjoiV2luMzIiLCJBTiI6Ik1haWwiLCJXVCI6Mn0%3D%7C2000%7C%7C%7C&sdata=bhD06mLLyPjCJkjBqeF7TGuVdeXEgpYbTcQQ7pxndXg%3D&reserved=0

For manuscripts utilizing custom algorithms or software that are central to the research but not yet described in published literature, software must be made available to editors and reviewers. We strongly encourage code deposition in a community repository (e.g. GitHub). See the Nature Portfolio guidelines for submitting code & software for further information.

Data

Policy information about availability of data

All manuscripts must include a data availability statement. This statement should provide the following information, where applicable:

- Accession codes, unique identifiers, or web links for publicly available datasets
- A description of any restrictions on data availability
- For clinical datasets or third party data, please ensure that the statement adheres to our policy

The data that support the findings of this study are available in the article and supplementary information. Source data are provided with this paper. Raw NGS data were deposited in SRA (BioProject: PRJNA933107, accession numbers: SRR23379932, rMERS-CoV v1691R Huh7 p10; SRR23379933, rMERS-CoV WT Huh7 p10) and can be accessed at this link: https://eur03.safelinks.protection.outlook.com/?url=https%3A%2F%2Fdataview.ncbi.nlm.nih.gov%2Fobject%2FPRJNA933107%3Freviewer%3Dfpbmt3cnd6620pg1pjgjgtn1nr&data=05%7C01%7CI.Sidorov%40lumc.nl%7Cf297df6d0c34449bf9b308db0b545aa4%7Cc4048c4fdd544cbd80495457aacd2fb8%7C0%7C0%7C638116229519224723%7CUnknown%7CTWFpbGZsb3d8eyJWIjoiMC4wLjAwMDAiLCJQIjoiV2luMzIiLCJBTiI6Ik1haWwiLCJXVCI6Mn0%3D%7C2000%7C%7C%7C&sdata=bhD06mLLyPjCJkjBqeF7TGuVdeXEgpYbTcQQ7pxndXg%3D&reserved=0

Human research participants

Policy information about studies involving human research participants and Sex and Gender in Research.

Reporting on sex and gender	N/A
Population characteristics	N/A
Recruitment	N/A
Ethics oversight	N/A

Note that full information on the approval of the study protocol must also be provided in the manuscript.

Field-specific reporting

Please select the one below that is the best fit for your research. If you are not sure, read the appropriate sections before making your selection.

☒ Life sciences ☐ Behavioural & social sciences ☐ Ecological, evolutionary & environmental sciences

For a reference copy of the document with all sections, see nature.com/documents/nr-reporting-summary-flat.pdf

Life sciences study design

All studies must disclose on these points even when the disclosure is negative.

Sample size	Sample size calculations were based on pilot studies performed in our laboratory to provide sufficient numbers of mice in each group and perform statistical testing. For all experiments, 4-30 mice per group were sufficient to detect differences between groups with a power value of 0.8 and a 5% significance threshold.
Data exclusions	No data points were excluded from data sets.
Replication	The growth curves in Figure 1A and B were performed twice and the virus replication studies in Supplementary Figure 1 were performed at least three independent times. Animal experiments were performed with 4-30 animals per group. The animal experiments in Figure 1C, Figure 2A and B and Figure 6B and C were performed twice while the rest of the animal/MERS-CoV challenge experiments were performed once. All attempts at replication were successful.
Randomization	The allocation of animals into experimental groups was random in all animal experiments. Animals were randomly assigned to cages with no additional knowledge of the study design. Mice were further separated according to their sex in order to achieve equal numbers of males and females in each group. Simple randomization of the animals was further carried with a dice thrown (e.g., below and equal to 3 for the for a specific group, over 3 for another group).<br>Due to the small sample size in experiments other than animal experiments, randomization was not performed and not commonly done in the field.
Blinding	Blind outcome assessment was applied for animal experiments were possible. However, the investigators were not blinded to the allocation of groups during the animal experiments. Blinding was not necessary because the results are quantitative and did not require subjective judgment or interpretation. For experiments other than animal experiments, blinding was not performed because the results are quantitative and did not require subjective judgement or interpretation.

Reporting for specific materials, systems and methods

We require information from authors about some types of materials, experimental systems and methods used in many studies. Here, indicate whether each material, system or method listed is relevant to your study. If you are not sure if a list item applies to your research, read the appropriate section before selecting a response.

Materials & experimental systems

n/a	Involved in the study
☐	☒ Antibodies
☐	☒ Eukaryotic cell lines
☒	☐ Palaeontology and archaeology
☐	☒ Animals and other organisms
☒	☐ Clinical data
☒	☐ Dual use research of concern

Methods

n/a	Involved in the study
☒	☐ ChIP-seq
☒	☐ Flow cytometry
☒	☐ MRI-based neuroimaging

Antibodies

Antibodies used	ELISPOT<br>BD Biosciences: CD3-BV510 (clone 145-2C11, cat 563024, 1:150) and CD28 (clone 37.51, cat 553294, 1:150)<br>IFN-γ ELISpot-plus kit 665, cat 3321-4AST-2 (Mabtech)<br>ELISA<br>ELISA kits 645 specific for mouse IFN-β (DY8234-05, R&D Systems), TNF-α (DY410-05, R&D Systems) , IL-6 (DY406-646 05, R&D Systems), IFN-λ(D485-05, R&D Systems), IFN-Y (DY1789B-05, R&D Systems), and IL-1β (D401-647 05, R&D Systems)
Validation	All antibodies used in this study are commercially available and have been validated by respective suppliers and their validation data are available on the manufacturers websites.<br>CD3-BV510 (clone 145-2C11, cat 563024, 1:150):<br>https://www.bdbiosciences.com/en-ca/products/reagents/flow-cytometry-reagents/research-reagents/single-color-antibodies-ruo/bv510-hamster-anti-mouse-cd3e.563024<br>CD28 (clone 37.51, cat 553294, 1:150):<br>https://www.bdbiosciences.com/en-us/products/reagents/flow-cytometry-reagents/research-reagents/single-color-antibodies-ruo/purified-na-le-hamster-anti-mouse-cd28.553294<br>IFN-γ ELISpot-plus kit 665, cat 3321-4AST-2 (Mabtech):<br>https://www.mabtech.com/products/elispot-plus-mouse-ifn-g-alp-3321-4ast<br>ELISA kits 645 specific for mouse IFN-β (DY8234-05, R&D Systems):<br>https://www.rndsystems.com/products/mouse-ifn-beta-duoset-elisa_dy8234-05<br>TNF-α (DY410-05, R&D Systems):<br>https://www.rndsystems.com/products/mouse-tnf-alpha-duoset-elisa_dy410<br>IL-6 (DY406-646 05, R&D Systems):<br>https://www.rndsystems.com/products/mouse-il-6-duoset-elisa_dy406<br>IFN-λ(D485-05, R&D Systems):<br>https://www.rndsystems.com/products/mouse-il-28b-ifn-lambda-3-duoset-elisa_dy1789b<br>IFN-Y (DY1789B-05, R&D Systems):<br>https://www.rndsystems.com/products/mouse-ifn-gamma-duoset-elisa_dy485<br>IL-1β (D401-647 05, R&D Systems):<br>https://www.rndsystems.com/products/mouse-il-1-beta-il-1f2-duoset-elisa_dy401

Eukaryotic cell lines

Policy information about cell lines and Sex and Gender in Research

Cell line source(s)	MRC-5, CCL-171, ATCC<br>Huh7, a kind gift of Dr. Ralf Bartenschlager, Heidelberg University, purchased from JCRB, No. JCRB0403<br>BHK-21 (C-13), CCL-10, ATCC
Authentication	BHK-21, MRC-5 and Huh7 cell lines were all commercially purchased and no additional authentication was performed.
Mycoplasma contamination	The cell-lines tested negative for Mycoplasma
Commonly misidentified lines (See ICLAC register)	Huh7, JTC-39

Animals and other research organisms

Policy information about studies involving animals; ARRIVE guidelines recommended for reporting animal research, and Sex and Gender in Research

Laboratory animals	hDPP4 KI mice (C57BL/6NTac-Dpp4tm3600(DPP4)Arte) were obtained from the National Center of Biotechnology (CNB-CSIC) Core Animal Facility, Campus Universidad Autónoma de Madrid, Madrid, Spain and bred in house. Male and female mice aged 8-12 weeks were used for the experiments.<br>Animals were housed in individually ventilated cages or in individually ventilated 554 isolator cages (IsoCage Biocontainment System, Tecniplast) under specific-pathogen free conditions at the animal facility at the Leiden University Medical Center (LUMC) at 20°C -22° C, a humidity of 45-65% RV and a light cycle of 6:30h-7:00h sunrise, 07:00h-18:00h daytime and 18:00h-18:30h sunset.
Wild animals	This study did not involve wild animals.
Reporting on sex	Both sexes of mice were used in this study based on literature research (Li et al. PNAS (2017)). For the age groups (8-12 weeks) used in this study, similar infection outcomes are expected for both male and female.
Field-collected samples	This study did not involve samples collected from the field.
Ethics oversight	All animal experiments were approved by the Animal Experiments Committee of LUMC and performed according to the recommendations and guidelines set by LUMC and by the Dutch Experiments on Animals Act.

Note that full information on the approval of the study protocol must also be provided in the manuscript.
